# A case report of agoraphobia following right parietal lobe surgery: changes in functional and structural connectivities of the multimodal vestibular network

**DOI:** 10.3389/fneur.2023.1163005

**Published:** 2023-05-12

**Authors:** Iole Indovina, Alberto Cacciola, Sergio Delle Monache, Demetrio Milardi, Francesco Lacquaniti, Nicola Toschi, Jerome Cochereau, Gianfranco Bosco

**Affiliations:** ^1^Brain Mapping Lab, Department of Biomedical and Dental Sciences and Morphofunctional Imaging, University of Messina, Messina, Italy; ^2^Laboratory of Neuromotor Physiology, IRCCS Santa Lucia Foundation, Rome, Italy; ^3^Departmental Faculty of Medicine and Surgery, Saint Camillus International University of Health and Medical Sciences, Rome, Italy; ^4^Department of Systems Medicine and Centre of Space BioMedicine, University of Rome Tor Vergata, Rome, Italy; ^5^Department of Biomedicine and Prevention, University of Rome “Tor Vergata”, Rome, Italy; ^6^Department of Radiology, Athinoula A. Martinos Center for Biomedical Imaging, Boston, MA, United States; ^7^Department of Neurosurgery, Poitiers University Medical Center, La Miletrie Hospital, Poitiers, France; ^8^Institute of Functional Genomics, INSERM 1191, University of Montpellier, Montpellier, France; ^9^University of Montpellier, Montpellier, France

**Keywords:** agoraphobia, graph theory, case report, connectivity, parietal glioma

## Abstract

Agoraphobia is a visuo-vestibular-spatial disorder that may involve dysfunction of the vestibular network, which includes the insular and limbic cortex. We sought to study the neural correlates of this disorder in an individual who developed agoraphobia after surgical removal of a high-grade glioma located in the right parietal lobe, by assessing pre- and post-surgery connectivities in the vestibular network. The patient underwent surgical resection of the glioma located within the right supramarginal gyrus. The resection interested also portions of the superior and inferior parietal lobe. Structural and functional connectivities were assessed through magnetic resonance imaging before and 5 and 7 months after surgery. Connectivity analyses focused on a network comprising 142 spherical regions of interest (4 mm radius) associated with the vestibular cortex: 77 in the left and 65 in the right hemisphere (excluding lesioned regions). Tractography for diffusion-weighted structural data and correlation between time series for functional resting-state data were calculated for each pair of regions in order to build weighted connectivity matrices. Graph theory was applied to assess post-surgery changes in network measures, such as strength, clustering coefficient, and local efficiency. Structural connectomes after surgery showed a decrease of strength in the preserved ventral portion of the supramarginal gyrus (PFcm) and in a high order visual motion area in the right middle temporal gyrus (37dl), and decrease of the clustering coefficient and of the local efficiency in several areas of the limbic, insular cortex, parietal and frontal cortex, indicating general disconnection of the vestibular network. Functional connectivity analysis showed both a decrease in connectivity metrics, mainly in high-order visual areas and in the parietal cortex, and an increase in connectivity metrics, mainly in the precuneus, parietal and frontal opercula, limbic, and insular cortex. This post-surgery reorganization of the vestibular network is compatible with altered processing of visuo-vestibular-spatial information, yielding agoraphobia symptoms. Specifically, post-surgical functional increases of clustering coefficient and local efficiency in the anterior insula and in the cingulate cortex might indicate a more predominant role of these areas within the vestibular network, which could be predictive of the fear and avoiding behavior characterizing agoraphobia.

## Introduction

Lesions in the posterior perisylvian cortex can be associated with several visuo-vestibular-spatial dysfunctions, such as spatial hemineglect syndrome (1, 2), pusher syndrome (3), and out-of-body experiences (4, 5). Another syndrome, potentially related to a visuo-vestibular-spatial disorder is agoraphobia (6), originally described as a condition of fear-related alterations in spatial orientation and locomotor control triggered by places or situations that might cause a patient to panic and feel trapped (7). Nowadays, this syndrome is considered largely of psychiatric interest and mostly related to panic attacks (8). Nonetheless, the visuospatial and vestibular components of agoraphobia should not be dismissed as increasing evidence indicates strong associations between vestibular and anxiety disorders (9–15). Indeed, the vestibular and anxiety systems do interact at multiple levels from the brainstem to the cortex (16). Moreover, functional MRI studies have highlighted that vestibular stimulation has profound effects on the activity and connectivity of both vestibular and anxiety-related brain regions, modulated by neuroticism and introversion (13–15). The basis for this extensive interaction may rest on the fact that vestibular information, unlike other sensory modalities, is not relayed directly to a classically defined “primary cortex,” but it engages a widely distributed network of brain areas integrating vestibular, visual, and somatosensory stimuli for the processing of motion and space. The main nodes of the vestibular network are represented by several regions around the parietal opercula, posterior insula, and adjacent perisylvian regions of the posterior parietal and temporal cortex (17–19), which also contribute significantly to the perception of gravity effects (20). The vestibular network extends further to the medial superior temporal area (MST), posterior inferior temporal gyrus, ventral intraparietal area, superior parietal lobe, sensory-motor cortex, hippocampal formation, anterior insula, inferior frontal gyrus, and cingulate cortex (20–24). Remarkably, some of these areas (e.g., anterior insula and hippocampus) are critically involved in emotional processing (25, 26), accounting for the close associations between anxiety and vestibular disorders observed clinically, including agoraphobia.

Along these lines, a recent resting-state functional MRI study reported that networks integrating visual, vestibular, and emotional signals to guide movement in space may be altered in subclinical agoraphobia (27). Moreover, agoraphobic patients show increased fMRI activation of the ventral striatum and insula when they expect agoraphobia-specific visual stimuli (28).

This study further investigated the neural correlates of this disorder in a patient who developed agoraphobia after surgical resection of a parietal glioma. Direct involvement of the parietal lobe in panic/agoraphobic symptoms has previously been advocated (29, 30). The area removed by surgery encompassed the right superior parietal lobule (5l, lateral area 5; 7PC, 7ip, postcentral, and intraparietal area 7), the intraparietal sulcus (hIP1/2/3, human intraparietal 1/2/3), and the inferior parietal lobe PFt (area supramarginalis tenuicorticalis), reaching inferiorly the anterior ventral supramarginal gyrus [named PIC in Indovina et al. (19)]. These areas can be considered hubs of the vestibular network and show significant decreases in topological network measures (local efficiency and clustering coefficient) in individuals with subclinical agoraphobia (19, 27). Based on this, it might be hypothesized that agoraphobia symptoms developed by this patient might have resulted from the reorganization of the connectivity within the vestibular network following the removal of these vestibular areas in the right hemisphere. We tested this idea by performing structural and functional connectivity analyses on MRI images acquired prior to and after the surgery using 142 spherical regions of interest defining bilaterally the visuospatial-emotional network (19). Consistent with the hypothesis, we found significant differences in several metrics of structural and functional connectivities.

## Methods

### Patient

We report the case of a 41-year-old female patient, left-handed, working as a salesperson, with no particular medical or psychiatric history, who presented a partial comitial seizure, characterized by transient agraphia. The patient gave written informed consent to all the procedures, which was approved by the local Ethical Committee. Following MR scan at 3T MAGNETOM Skyra (Siemens Medical Systems, Erlangen, Germany) at La Miletrie Hospital (Poitiers University Medical Center, Poitiers, France), a FLAIR hypersignal localized in the superior portion of the right supramarginal gyrus was identified. Anti-comitial treatment with levetiracetam was initiated (1,000 mg twice a day) and, in view of the strong suspicion of diffuse glioma, the patient underwent surgery. The procedure was performed according to the local protocol, i.e., an asleep/awake/asleep surgery with positive cortical mapping without electrophysiology recordings. Subcortical stimulations near the posterior insular cortex caused dizziness sensations. A post-operative MR scan confirmed the supra-total resection of the FLAIR anomaly. Post-operative follow-up was marked by rapidly resolving praxis difficulties and agraphia. However, as soon as she returned home, the patient reported symptoms suggestive of agoraphobia, leading to avoidance behavior.

The outcomes of psychiatric and cognitive tests performed prior to and after the surgery are reported in the Supplementary material.

### MRI acquisitions

MRI images were acquired at three different times: pre-surgery (pre-op) and 5 months (post-op1) and 7 months after surgery (post-op2) on a 3T MAGNETOM Skyra (for details see Supplementary material).

### Gray matter parcellation and location of the lesion

To identify the areas removed by surgery, we realigned the anatomical acquisitions to the MNI 152 T1 1 mm template with the ANTs toolbox (31).

**Figure 1:**
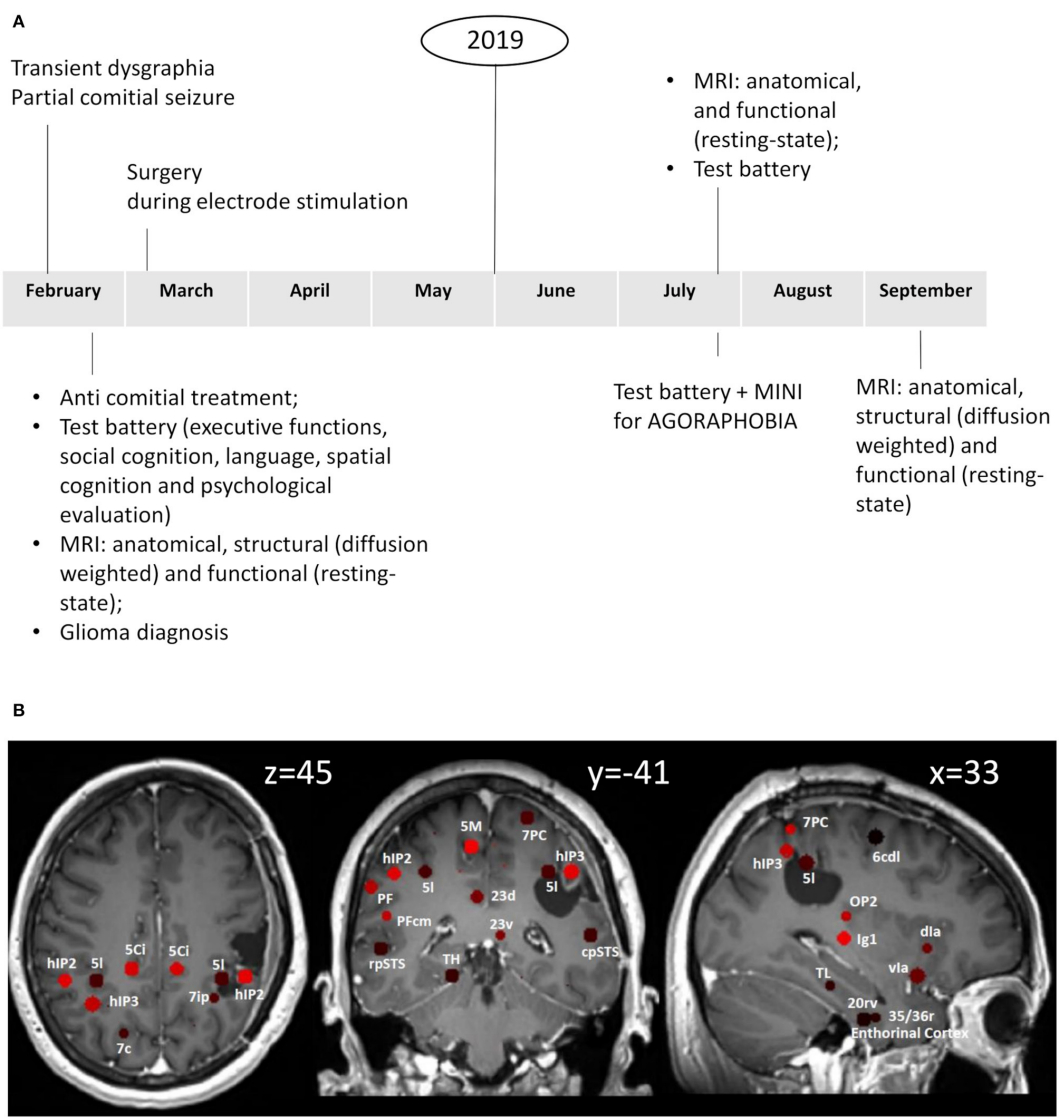
**(A)** Timeline with relevant episodes of care. **(B)** Sphere atlas superimposed on the first anatomical image taken after surgery. The anatomical image was realigned to the MNI 152 ICBM 2009a_nlin_hd_1mm template through ANTs.

We used a “Sphere atlas” described previously (19) to define nodes for the construction of functional (from resting-state data)- and structural (from DWIdata)-weighted connectivity matrices. This atlas consisted of spherical regions (4 mm radius) placed on the geometric centers of regions defined in the Eickhoff (32) and Fan (33) atlases [see (19) for details]. We limited our analysis to 77 regions belonging to the vestibular network (Table 1). To identify and exclude the areas lying within the surgical bed, we overlapped the anatomical image acquired after surgery with the Sphere atlas (Figure 1). Details on the functional imaging and graph analyses are reported in the Supplementary material.

**Table 1:** A total of 77 selected regions in the multimodal vestibular network.

**Location**	**Label**	**Area**
Inferior Frontal gyrus	44d	Dorsal area 44
	44op	Opercular area 44
	44v	Ventral area 44
	44	Caudal area 44
	45	Rostral area 45
	45c	Caudal area 45
Precentral gyrus	4tl	Area 4 (tongue and larynx region)
	6cdl	Caudal dorsolateral area 6
	6cvl	Caudal ventrolateral area 6
Postcentral gyrus	1/2/3/tonla	1/2/3 tongue, larynx
Middle and superior temporal gyrus	MT/MST	Visual motion complex
	37dl	Dorsolateral area37
	37vl	Ventrolateral area 37
	37mv	Medioventral area 37
	37lv	
	cpSTS	Caudal posterior superior temporal sulcus
	rpSTS	Rostral posterior superior temporal sulcus
Inferior temporal gyrus	20rv	Rostroventral area 20
Parahippocampal gyrus	Entorhinal Cortex	
	35/36c	Caudal area 35/36
	35/36r	Rostral area 35/36
	28/34	Area 28/34
	TL	Area TL (lateral posterior parahippocampal gyrus)
	TH	Area hippocampotemporalis
Hippocampus proper	Subiculum	
	cHipp	Caudal hippocampus
	rHipp	Rostral hippocampus
Insula	Id1	Dysgranular insula
	Ig1	Granular insula 1
	Ig2	Granular insula 2
	dIa	Dorsal agranular insula
	dId	Dorsal dysgranular insula
	dIg	Dorsal granular insula
	vIa	Ventral agranular insula
	vId/vIg	Ventral dysgranular and granular insula
	TI	Area TI (temporal agranular insular cortex)
Parietal operculum	OP1	Secondary somatosensory area (SII)
	OP2	Parieto insular vestibular cortex (PIVC)
	OP3	Ventral somatosensory area (VS)
	OP4	Parietal ventral area (PV)
Cingulate gyrus	23c	Caudal area 23
	23d	Dorsal area 23
	23v	Ventral area 23
	24cd	Caudodorsal area 24
	24rv	Rostroventral area 24
	32p	Pregenual area 32
	32sg	Subgenual area 32
Inferior parietal cortex	**PF**	Area supramarginalis
	PFcm	Area supramarginalis columnata magnocellularis (posterior)
	**PFm**	Area supramarginalis magnocellularis
	Pfop	Area supramarginalis opercularis
	**PFt**	Area supramarginalis tenuicorticalis
	**PIC**	Rostroventral area 40
	39rv	Rostroventral area 39
Intraparietal sulcus	**hIP1**	Human intraparietal 1
	**hIP2**	Human intraparietal 2
	**hIP3**	Human intraparietal 3
Superior parietal lobe	5Ci	
	5L	
	**5l**	Lateral area 5
	7A	Medial area 7
	7P	
	7c	Caudal area 7
	**7ip**	Intraparietal area 7
	**7PC**	Postcentral area 7
	7pc	
	7r	Rostral area 7
Precuneus	5M	Medial area 5
	7M	Medial area 7
	7m	Medial area 7
	dmPOS	Dorsomedial parieto-occipital sulcus
	31	Area 31
Thalamus	Thal Parietal	
	Thal Premotor	
	Thal Temporal	
Cerebellum	Fastigial Nuclei	

The 10 areas in bold are those within the lesion that are excluded from the analysis (5l, 7ip, 7PC, PIC, hIP1, hIP2, hIP3, PF, PFt, and PFm, only in the right hemisphere).

## Results

### Structural connectivity

**Figure 2:**
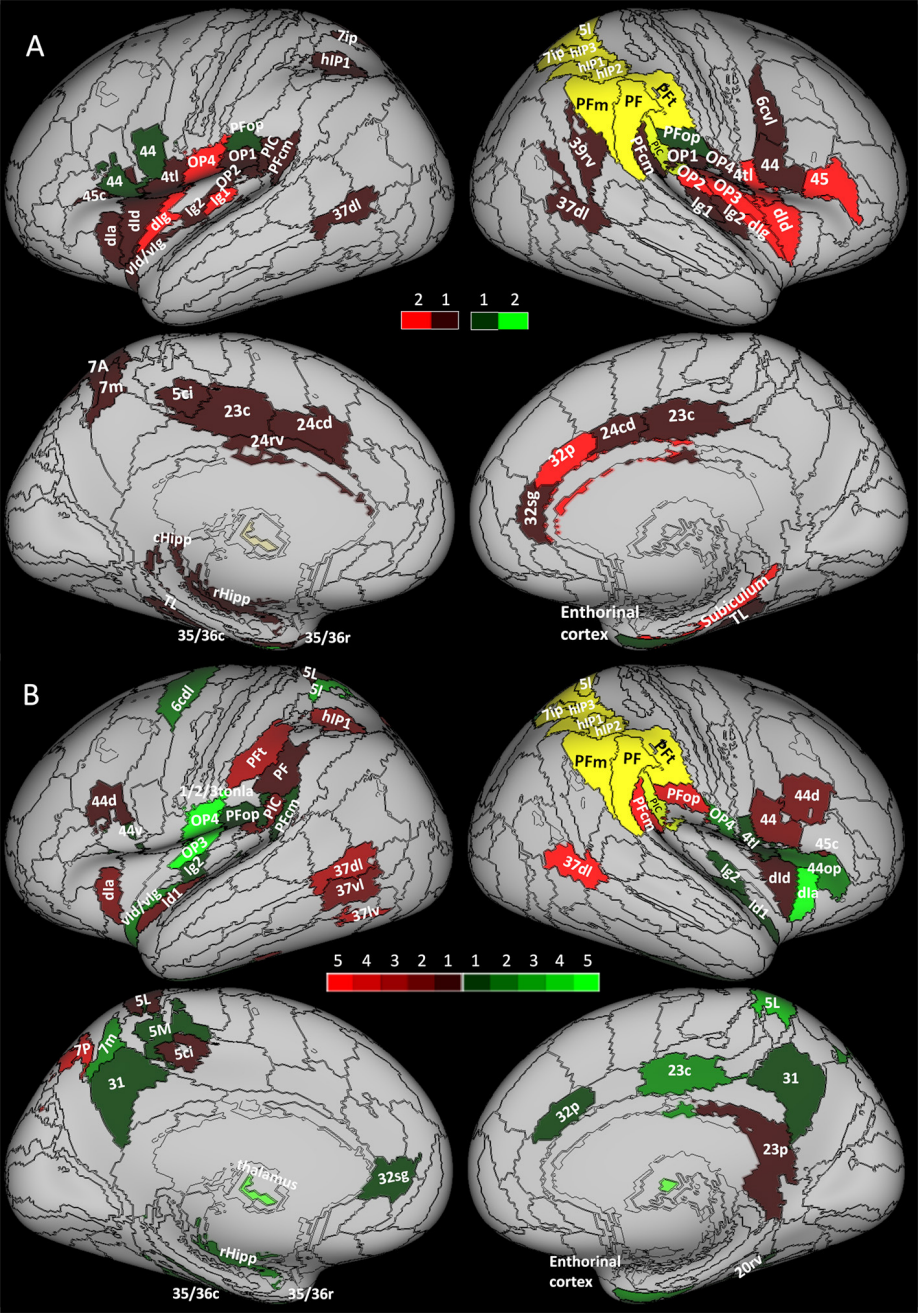
Increase (green) and decrease (red) of network measures between **(A)** structural sessions and **(B)** functional sessions. The yellow area represents the areas excluded from the analysis due to the surgical resection. **(A)** Brighter areas show changes in both clustering coefficient and local efficiency during pre-op session vs. post-op 2. The darkest areas show changes only in the clustering coefficient. **(B)** Brighter areas show changes in several comparisons in strength, cluster coefficient, and local efficiency (during pre-op vs. post-op 1 and pre-op vs. post-op2). The darkest areas show changes in only one comparison.

For each node of the network (see Table 1), we compared the nodal strength, clustering coefficient, and local efficiency measured before (pre-op session) and 7 months after surgery (post-op2 session). Although none of the network areas showed significant changes in connection strength, the network did show a general reorganization reflected by significant changes in the clustering coefficient and local efficiency. As Figure 2A shows, the majority of areas (*n* = 54, red) showed a decrease in the clustering coefficient. These 54 areas were located in the limbic cortex (hippocampal and parahippocampal cortex, cingulate gyri), bilateral insula, parietal opercula and inferior parietal cortex, left superior parietal cortex, right middle temporal gyrus, and right premotor cortex (see also Supplementary Figure). Only five areas (green in Figure 2A), mainly located in the parietal and limbic cortex, showed an increase in the local clustering coefficient.

A subset of those areas (*n* = 15) showing a decrease in clustering coefficient (Figure 2A, brighter red areas) showed also a decrease in the local efficiency, indicating a lower degree of communication between the nodes' neighbors. These areas belonged to the insula, parietal opercula, and right BA 4tl and 45.

Overall, the results of the structural connectivity analysis indicated a general decrease in the local network connectivity in the limbic cortex (insula, hippocampal formation, and cingulate cortex), the parietal opercula, the area 44, and high-order visual areas.

### Functional connectivity

For each node of the network, we compared the connectivity metrics before surgery with that observed 5 and 7 months after surgery (pre-op, post-op1, and post-op2 sessions, respectively).

#### Pre-op vs. post-op1

As illustrated in Figure 2B, two regions in the middle/superior temporal gyrus of the right hemisphere (dorsolateral BA 37, 37dl) and in the supramarginal gyrus (PFcm) showed a significant decrease in connectivity strength, clustering coefficient, and local efficiency.

Additional areas showed reduced clustering coefficient, such as other high-order visual areas and the superior parietal cortex in the left hemisphere. In a subset of these areas, clustering coefficient decreases were accompanied by decreases in local efficiency.

Conversely, the clustering coefficient increased significantly in the right entorhinal cortex, the right inferior temporal gyrus, the left postcentral gyrus, as well as bilaterally in the parietal opercula, the insula, the precuneus, and the cingulate cortex. Again, in a subset of these regions, local efficiency was also increased.

#### Pre-op vs. post-op2

With this comparison, we noted that the decrease in connectivity strength observed 5 months after the surgery in the right PFcm and in the right 37dl persisted. Similarly, in the inferior parietal cortex and opercula bilaterally, in bilateral high-order visual areas, and in the left superior parietal lobule (see Figure 2B, Supplementary Figure), the reduction in the clustering coefficient was also evident at post-op2. Other cortical areas that did not show changes in post-op1, such as the premotor cortex and the anterior insula bilaterally, showed a reduction in the clustering coefficient in post-op2, mimicking the findings of the structural analysis performed on data drawn from the same sessions. Moreover, local efficiency was reduced in several of these regions.

Finally, a good degree of overlap with the pre-op vs. post-op1 comparison was also evident for the network nodes showing increased clustering coefficient and local efficiency.

In summary, unlike the structural connectivity analysis that showed a general decrease in the local network connectivity, functional analysis showed that the nodal clustering coefficient and local efficiency could decrease in some areas, notably high-order visual areas and parietal cortex, while increasing in some other areas, particularly the medial superior parietal cortex (precuneus) and opercula, hippocampal/parahippocampal cortex, and the insular cortex. Moreover, in some areas (right entorhinal cortex, left vId/vIg), significant changes were evident in the first post-surgical session and partially reverted in the second session.

In other words, the results of the structural and functional analyses overlapped only partially. On the one hand, a good degree of overlap was evident, for example, in high-order visual 37dl, inferior parietal cortex (PFcm and rostral supramarginal gyrus PIC), and premotor areas, which showed a general decrease in both structural and functional connectivities. On the other hand, the two analyses showed divergent results in the limbic cortex where a general decrease in structural connectivity metrics was accompanied by a significant increase in functional connectivity metrics. Finally, other areas, such as the right anterior insula and frontal operculum, the subgenual cingulate, the bilateral precuneus, the left OP3, and the left 6cdl, showed an increase only in functional connectivity without significant changes in structural connectivity.

## Discussion

Herein, we report the case of a patient who developed agoraphobic symptoms after surgical resection of a right parietal glioma. Glioma patients may experience impairments in motor and non-motor activities prior to treatment and are inherently at increased risk for further psychomotor, visuospatial, and emotional decline after surgery. While according to the localizationist theory, the anatomical location of tumors can predict specific symptoms, increasing evidence suggests that neuropsychiatric impairments may emerge from the disruption of more complex networks involving brain areas distributed both in close proximity to and far from the lesion (34–40).

This is of relevance, especially for gross total or supramaximal resections, in which the extent of resection exceeds any visible abnormalities. In this case report, the extent of surgery encompassed the right superior parietal lobule, the intraparietal sulcus, and the inferior parietal lobe, reaching inferiorly to the anterior ventral supramarginal gyrus. Considering the connectivity patterns of these areas and their involvement in the multimodal vestibular network, it may be reasonable to assume that the development of agoraphobic symptoms could derive from the disconnection caused by the resection and/or by adaptive compensatory mechanisms leading to abnormal network reorganization.

**Figure 3:**
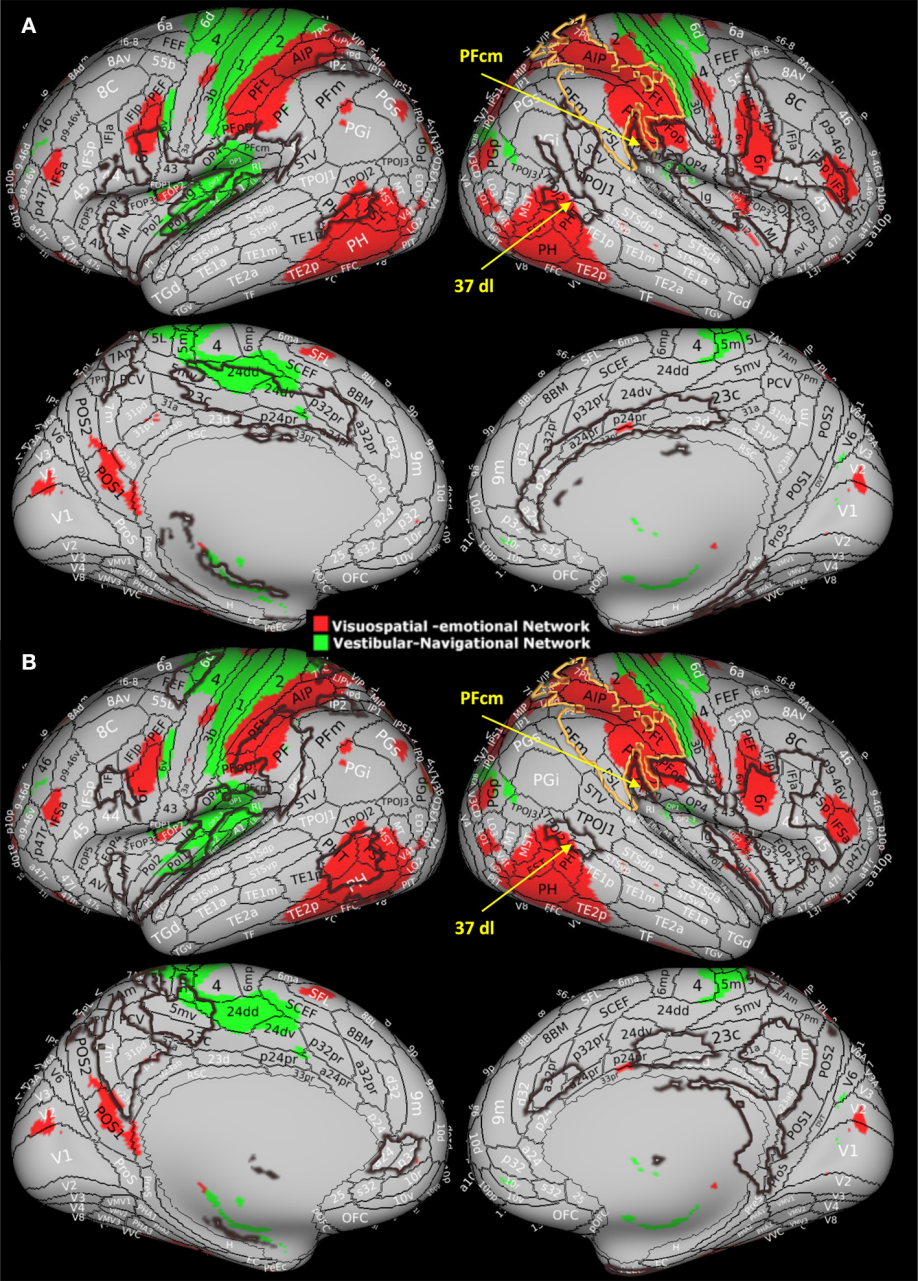
Visuospatial-emotional network (red) and vestibular-navigational network (green) from Indovina et al. (27) study. These networks showed reduced clustering coefficient and local efficiency in individuals with subclinical agoraphobia compared to controls. In this study, 37dl corresponds to PHT by von Economo and Koskinas (41, 42). Modified with permission from Indovina et al. (27) by superimposing current results, i.e., the area of surgical resection (yellow contour) and **(A)** areas presenting structural measures alterations and **(B)** areas presenting functional measures alterations (brown contours).

We previously hypothesized that agoraphobia is related to dysfunction of the multimodal vestibular network that relates visuospatial processing to the emotional system (27) based on the finding that subclinical agoraphobia was associated with decreases in the clustering coefficient and in the local efficiency in two specific networks highly overlapping with the vestibular network (Figure 3). The first network, considered visuospatial-emotional, comprised higher-order visual and visual motion areas, the inferior parietal cortex, the precuneus, premotor areas, the anterior thalamus, the basal ganglia, and the amygdala (Figure 3, red). The second network, defined as vestibular-navigational, comprised the hippocampus, posterior regions of the insula and parietal opercula, the superior parietal cortex, the primary somatosensory and motor cortex, and premotor regions (Figure 3, green). Together, these two networks integrate visual information, particularly visual motion stimuli generated during self-motion and processed by high-order temporal visual areas, with vestibular and somatosensory information from the posterior insula and parietal opercula to orient the body in space (parietal cortex) and to map the environment (hippocampus). Frontal regions within these networks (premotor and pre-frontal regions and cingulate cortex) elaborate this information to plan and either initiate or inhibit locomotion, which could be driven also by motivation and/or environmental threats processed by the amygdala, hippocampus, and associated subcortical structures. Thus, agoraphobia may not be related to the dysfunction of isolated brain regions, but it may involve the disruption of wider networks responsible for mapping the environment and driving the intention to interact with it (27).

Here, we provide further evidence that extensive post-surgery reorganization within the vestibular network, signified by the structural and functional connectivity metric changes shown by several network nodes, could account for the agoraphobic symptoms reported by this patient. Notably, the surgical lesion is prominently located within the visuospatial-emotional network (Figure 3, yellow contours), suggesting that, at least in part, the development of the agoraphobic symptoms could be accounted for by the surgical disruption of this network component *per se*. Moreover, both visuospatial-emotional and vestibular-navigational networks highly overlapped with regions that showed significant structural (Figure 3A, brown contours) and functional (Figure 3B, brown contours) connectivity changes. In particular, the high-order visual right 37dl (dorsolateral Brodmann area 37) and right PFcm in the inferior parietal lobe showed a generalized decrease in all connectivity metrics, including functional nodal strength (see Figure 2B). These areas are considered to be involved in navigation and orienting (22) and are also part of the visuospatial-emotional network that showed decreased connectivity in subclinical agoraphobia (Figure 3). Thus, plastic reorganization of the visuospatial-emotional network, consisting of a local disconnection of these areas, could have also contributed to the development of agoraphobia in this patient.

Conversely, regions that showed an increase in functional connectivity metrics and can be grouped in an attentional and action-reorienting network, such as the anterior insula, anterior cingulate cortex, and precuneus, do not show a great degree of overlap with the networks emerging in subclinical agoraphobia. Among these, the precuneus is involved in visual-spatial guided behavior, attentive tracking, and attentional shifting (43, 44), while the right anterior insula and the frontal operculum are associated with the exogenous ventral attention network, which is lateralized to the right hemisphere and can monitor environmental threats (45), initiating the sympathetic fight or flight response (26). In particular, the anterior insula integrates interoceptive information with emotional feelings, whereas the anterior cingulate cortex regulates the sympathetic response accompanying the avoidance or approaching behavior (26). It has also been proposed that these right hemisphere regions are involved in the appraisal of negative feelings and avoidance behavior, respectively, in contrast to the opposite role of the homolog contralateral areas in positive feelings appraisal and approaching behavior (26). In this respect, the post-surgical functional increase in clustering and efficiency of the anterior insula and cingulate cortex might be predictive of the fear of surrounding spaces and avoiding behavior, which is characteristic of agoraphobia. The involvement of these regions outlined by the current study might also account for the difference between subclinical and clinical agoraphobia.

On the other hand, no definite conclusions can be drawn from our results about a potential laterality bias since the cingulate cortex and the anterior insula showed mixed bilateral increases and decreases in the functional connectivity metrics. Moreover, the patient is left-handed, thus, possibly presenting a different pattern of lateralization, as left-handers, compared to right-handers, may show a more symmetrical organization of lateralized functions rather than opposite hemisphere lateralization (46). Indeed, recovery from a left-hemispheric stroke seems more rapid and complete in left-handers than in right-handers with right-hemispheric stroke, thus suggesting higher bilateral processing of vestibular stimuli in left-handers than right-handers (47, 48). Based on these considerations, we may hypothesize that the patient's left-handedness had uncovered more subtle symptoms than the typical clinical picture of the visuospatial hemi-neglect manifested by patients with right parietal lesions.

## Conclusion

We provided evidence that extensive reorganization of the multimodal vestibular network could account for the development of agoraphobic symptoms in a patient after the surgical removal of a right parietal glioma. Specifically, the decrease in the clustering coefficient and in the local efficiency observed with both structural and functional connectivity analyses in several areas of the limbic, insular, parietal, and frontal cortex was strongly indicative of a local disconnection of these regions belonging to the vestibular network. Conversely, the post-surgical functional increases in clustering coefficient and local efficiency in the anterior insula and in the cingulate cortex indicated a more predominant role of these areas within the vestibular network, which could be predictive of the fear and avoiding behavior characterizing agoraphobia.

## Data availability statement

The raw data supporting the conclusions of this article will be made available by the authors, without undue reservation.

## Ethics statement

The studies involving human participants were reviewed and approved by Poitiers University Hospital. The patients/participants provided their written informed consent to participate in this study. Written informed consent was obtained from the individual(s) for the publication of any potentially identifiable images or data included in this article.

## Author contributions

II and AC: conceptualization, data analysis, and wrote the manuscript. SD, DM, and FL: edited the manuscript. NT: data analysis. JC: data acquisition and wrote the manuscript. GB: conceptualization and wrote the manuscript. All authors contributed to the article and approved the submitted version.
