# Supplementary material for: A case report of agoraphobia following right parietal lobe surgery: changes in functional and structural connectivities of the multimodal vestibular network
Source: Front Neurol. 2023 May 12;14:1163005. doi: 10.3389/fneur.2023.1163005 (PMC10213528; doi:10.3389/fneur.2023.1163005)
Supplement: Supplementary file 1 [file Data_Sheet_1.docx]

## **Supplementary Methods**

## **Patient**

41 years old women, married, no children, no past medical history, working as a salesman.

After the partial seizure, she said she was bothered by noise and light, and had increased irritability.

Keppra was introduced preoperatively at the dose of 1000 mg twice a day. Before surgery she presented two partial motor seizures (left hand tremor) lasting about ten seconds.

Before surgery she also described feelings of floating, she had difficulties in naming, and she presented dysgraphic elements.

**Intervention**

The intervention consisted in a glioma resection by the mean of intraoperative stimulation brain mapping (cortical and sub-cortical stimulations) with the aim of achieving maximal tumor removal (supra-total resection) without lasting neurological deficits (De Witt Hamer et al., 2012). Glioma resection was performed after positive cortical mapping eliciting primary somato-motor cortex responses. It is worth mentioning that at the end of the resection, sub-cortical stimulations at the postero-superior aspect of the insula caused vertigo.

That surgery was justified because supra-total resection of the FLAIR hyperintensities was achievable according to the preoperative imaging which has a high statistical impact on diffuse glioma prognosis (Jakola et al., 2017).

## **Outcomes of psychiatric and cognitive tests**

The MINI-S for DSM-V battery [French version (Sheehan et al., 1998)] performed at post-op1 after surgery confirmed the agoraphobia diagnosis without panic disorder based on the following symptoms: avoidance of places or situations where it might be difficult to escape from or finding help leading her to consistently seek company when such situations could not be avoided; marked fear when confronted to closed spaces such as shops, cinemas, theaters, and fear of waiting lines.

The patient was also administered a series of tests three days before surgery and at post-op1: “Batterie d’evaluation de la négligence spatiale”, BEN – unilateral neglect evaluation battery (Geren 2000) (Azouvi et al., 2006); hospital anxiety depression scale, HADS (Zigmond and Snaith, 1983); global cognitive efficiency test [evaluation rapide des fonctions cognitives, ERFC, (GIL et al., 1986)] and evaluation of memory and executive functions (WAIS-IV) [Processing Speed, Working Memory Index, Wechsler, D. (2008a). Wechsler Adult Intelligence Scale – Fourth Edition. San Antonio, TX: Pearson Assessment.

The Geren 2000 showed outside normal range parameter only in the post-op1 clock drawing time (117 s post-op1 vs 37 s pre-op, normal values < 70 s).

According to the HADS questionnaire, the patient exhibited clear profiles for anxiety (HADS pre: 12 and post-op1: 13) and depression (HADS pre: 8 post-op1: 12) both pre- and post-surgery.

The patient scores were normative in the global cognitive efficiency test (ERFC) and evaluation of memory and executive functions (WAIS-IV).

**Patient perspective**

Remotely from surgery (2023), the patient continues to describe a social life that is very different from what it was before with a more solitary behavior without this being experienced in a painful way. She is currently undergoing professional retraining. The resection profoundly modified the patient's social relationships and it is therefore very likely that the damage to the multimodal vestibular network could not be compensated.

**MRI acquisitions.**

**Functional data.** Resting-state EPI-BOLD images were acquired (TR: 2.56 s, TE: 30 ms, Flip angle: 90, 448 x 448, Voxel size 3 x 3 x 3 mm, Slice spacing: 3.3 mm, Number of volumes: 250, Number of slices: 44, Pixel-Bandwidth: 2440) at pre-op, post-op1 and post-op2. During scanning, the participant was instructed to rest, but to remain awake with her eyes closed.

**Structural data.** Diffusion weighted images (DWI) were acquired only at pre-op and post-op2 with a 64 channels head and neck coil using the following parameters: Voxel size 2 x 2 x 2 mm, TE: 0.106 s, TR: 5.5 s, Flip Angle: 90 degrees, Partial Fourier: 0.75, Diffusion Scheme: Bipolar, Multiband Acceleration Factor: 2. One unweighted b0 volume and 64 diffusion encoding directions were acquired. The *b*-value was set to 1000 s/mm^2^.

Three anatomical MPRAGE images were also acquired.

**Analysis**

**Structural imaging analysis.** DWI preprocessing included denoising (Veraart et al., 2016) with the MRtrix3 dwidenoise command (Tournier et al., 2019), and eddy current and motion correction through the ‘EDDY’ tool [MRtrix3 dwifslpreproc, that uses FSL (Jenkinson et al., 2012)]; B1 field inhomogeneity correction was performed through dwibiascorrect (MRtrix3). Starting from these preprocessed data, Tournier iterative algorithm for single-fibre voxel selection and response function estimation (Tournier et al., 2013), followed by probabilistic tractography and spherical-deconvolution informed filtering of tractograms (SIFT) (Smith et al., 2013) was used to estimate whole-brain tractograms (Tournier et al., 2007) in MRtrix3. We employed the probabilistic tractography by 2^nd^ order integration over fiber orientation distributions (iFOD2) algorithm (Willats et al., 2014). 10^7^ fibers streamlines were generated by randomly seeding from the white matter, and then filtered using SIFT by a factor 10 to obtain more precise anatomical correspondence in the final set of 10^6^ streamlines.

In order to precisely match the “Sphere atlas” to the individual space in which tractography was performed, the T1 image was firstly coregistered to the DWI, and the resulting coregistered T1 was later normalized to the MNI T1 template (MNI 152 ICBM 2009a_nlin_hd_1mm) using high rigid, affine and deformable registration within the ANT’s symmetric diffeomorphic nonlinear image registration tool (SyN) (Avants et al., 2011). The inverse transform was applied to the atlas in order to project it into the single subject space. Weighted connectivity matrices for the pre-op and the post-op2 were constructed by combining the tractograms with the sphere atlas in the native space (77 nodes for the left hemisphere and 65 nodes for the right hemisphere, excluding regions removed surgically), normalizing the number of streamlines for each pairwise connection by the total volume of the two nodes. No thresholding on the weighted connectivity matrices was applied (Civier et al., 2019).

**Functional imaging analysis.** Resting-state fMRI images were preprocessed using the Matlab based platform “CONN” and SPM12 (https://www.fil.ion.ucl.ac.uk/spm/). Preprocessing pipeline included realignment, coregistration and normalization to the MNI template, and smoothing using an 8 mm Gaussian kernel.

In order to remove possible sources of confound and noise from the BOLD signal before computing connectivity measures, a denoising procedure was run. This analysis removed confounds deriving from BOLD signal from the white matter and CSF masks, and estimated subject motion parameters.

Then, for each session, a weighted connectivity matrix representing a functional brain network was computed. In this context, nodes were represented by the brain regions in the “Sphere atlas” (see Table 1) and edges were defined as the interregional functional connectivity. More precisely, network edges were defined calculating pairwise Pearson correlation coefficients between each pair of denoised time-courses.

This step resulted in three 142x142 symmetric and weighted matrices, one for each session (77 nodes for the left hemisphere and 65 nodes for the right hemisphere, by excluding the nodes in the lesioned area). All negative correlations were set to zero.

**Graph analysis**

In order to detect changes in the structural and functional connectivity across the three sessions, three metrics were computed for each ROI using the Brain Connectivity Toolbox (Rubinov and Sporns, 2010). Specifically, we focused on the nodal strength, clustering coefficient and local efficiency. Nodal strength is the sum of weights of edges connecting to that node, while local clustering coefficient and local efficiency are similar measures of connectivity of the neighbors of the node between them, thus reflecting the presence of a “cluster” (Rubinov and Sporns, 2010).

In particular, a node’s clustering coefficient is directly related to the number of triangles that have the node as a vertex. On the other hand, changes in local efficiency are directly related only to the connectivity of the node's neighbours. It has been asserted that if a node has high local efficiency, its neighbours may replace this node in case of loss, and thus local efficiency would reflect replaceability of the node.

Differences were calculated for each couple of sessions: (i) pre-op vs post-op1; (ii) pre-op vs post-op2; and (iii) post-op1 vs post-op2.

To test for significant differences in the selected metrics (strength, clustering coefficient and local efficiency) of each ROI across sessions, a set of 10000 pairs of random networks preserving the nodal degree (Iorio et al., 2016) were generated and a distribution of differences between each of the metrics for each node was computed. A p-value for each brain region was calculated as the fraction of differences exceeding the actual difference. A false discovery rate procedure (FDR) was used to correct for multiple comparisons over the brain areas. The significance threshold was set at p < 0.05 FDR corrected.

**
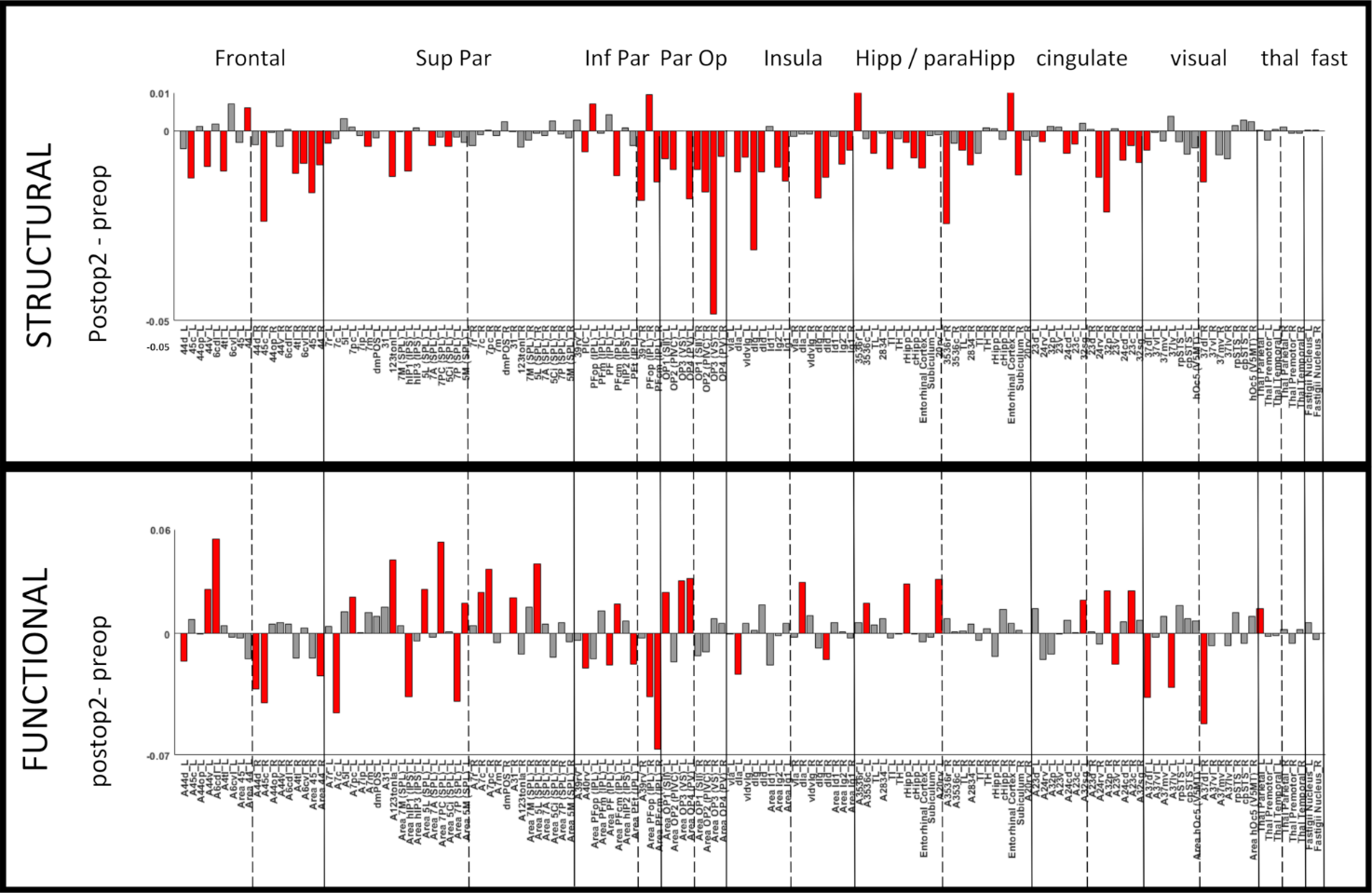
**

**Supplementary Figure.** Structural and functional changes in clustering coefficient in **postop2 vs preop**. Red bars indicate regions with statistically significant differences (p<0.05 FDR corrected). Vertical continuous lines separate macro-regions while dotted lines separate regions on the left from regions on the right. Sup Par: Superior parietal regions; Inf Par: Inferior parietal regions; Par Op: Parietal operula; Hipp/ParaHipp: hippocampal and para-hippocampal regions; thal: thalamus; fast: fastigial nuclei.

**Additional Results**

**Post-op1 vs post-op2.** When comparing the two post-surgical sessions, we found significant clustering coefficient reductions in motor and premotor cortex (right 4tl, right 45c, 44 bilateral), in the left 7m, in the limbic cortex (bilateral insula, right parahippocampal 20rv, entorhinal cortex and 35/36), which contrasted the findings of the pre-op vs post-op1 comparison.

Clustering coefficient increases were evident in several limbic regions including cingulate areas (right 32p and 23c, left 32sg), hippocampal and parahippocampal regions (left rHipp, left 20rv) and left insula (Id1), as well as in the bilateral superior parietal cortex, visual areas (right MT, left rpSTS, left 37vl) and left premotor areas (44v, 6cdl).

The local efficiency decreased in the left ventral insula (vId/vIg), in the right Area 44 and 44tl, whereas it increased in the superior parietal lobe (left 5L, right 7pc), left 37vl and left 6cdl.

**Limitations of the study:**

The main aim of this study was understanding the pathophysiology of agoraphobia, by characterizing the changes in the connectivity of vestibular network areas and relating them to the development of agoraphobia. While this approach has been very informative for the case study presented here, it should be substantiated by more extensive population studies on agoraphobic patients to draw more definite conclusions. Another limitation of the study regards its direct application to the clinical setting, in terms of delivering effective treatment of the agoraphobia symptoms, even though a better characterization of the involvement of the vestibular network may pave the way to novel therapeutic approaches.

**References**

Avants, B. B., Tustison, N. J., Song, G., Cook, P. A., Klein, A., and Gee, J. C. (2011). A reproducible evaluation of ANTs similarity metric performance in brain image registration. *Neuroimage* 54, 2033–2044. doi: 10.1016/j.neuroimage.2010.09.025.

Azouvi, P., Bartolomeo, P., Beis, J.-M., Perennou, D., Pradat-Diehl, P., and Rousseaux, M. (2006). A battery of tests for the quantitative assessment of unilateral neglect. *Restor Neurol Neurosci* 24, 273–285.

Civier, O., Smith, R. E., Yeh, C.-H., Connelly, A., and Calamante, F. (2019). Is removal of weak connections necessary for graph-theoretical analysis of dense weighted structural connectomes from diffusion MRI? *NeuroImage* 194, 68–81. doi: 10.1016/j.neuroimage.2019.02.039.

De Witt Hamer, P. C., Robles, S. G., Zwinderman, A. H., Duffau, H., and Berger, M. S. (2012). Impact of intraoperative stimulation brain mapping on glioma surgery outcome: a meta-analysis. *J Clin Oncol* 30, 2559–2565. doi: 10.1200/JCO.2011.38.4818.

GIL, R., TOULLAT, G., PLUCHON, C., MICHENEAU, D., CARIOU, B., RIVAULT, L., et al. (1986). Une méthode d’évaluation rapide des fonctions cognitives (ERFC). Son application à la démence sénile de type Alzheimer. *Sem. hôp. Paris* 62, 2127–2133.

Iorio, F., Bernardo-Faura, M., Gobbi, A., Cokelaer, T., Jurman, G., and Saez-Rodriguez, J. (2016). Efficient randomization of biological networks while preserving functional characterization of individual nodes. *BMC Bioinformatics* 17, 542. doi: 10.1186/s12859-016-1402-1.

Jakola, A. S., Skjulsvik, A. J., Myrmel, K. S., Sjåvik, K., Unsgård, G., Torp, S. H., et al. (2017). Surgical resection versus watchful waiting in low-grade gliomas. *Ann Oncol* 28, 1942–1948. doi: 10.1093/annonc/mdx230.

Jenkinson, M., Beckmann, C. F., Behrens, T. E. J., Woolrich, M. W., and Smith, S. M. (2012). FSL. *Neuroimage* 62, 782–790. doi: 10.1016/j.neuroimage.2011.09.015.

Rubinov, M., and Sporns, O. (2010). Complex network measures of brain connectivity: uses and interpretations. *Neuroimage* 52, 1059–1069. doi: 10.1016/j.neuroimage.2009.10.003.

Sheehan, D. V., Lecrubier, Y., Sheehan, K. H., Amorim, P., Janavs, J., Weiller, E., et al. (1998). The Mini-International Neuropsychiatric Interview (M.I.N.I.): the development and validation of a structured diagnostic psychiatric interview for DSM-IV and ICD-10. *J Clin Psychiatry* 59 Suppl 20, 22-33;quiz 34-57.

Smith, R. E., Tournier, J.-D., Calamante, F., and Connelly, A. (2013). SIFT: Spherical-deconvolution informed filtering of tractograms. *Neuroimage* 67, 298–312. doi: 10.1016/j.neuroimage.2012.11.049.

Tournier, J.-D., Calamante, F., and Connelly, A. (2007). Robust determination of the fibre orientation distribution in diffusion MRI: non-negativity constrained super-resolved spherical deconvolution. *Neuroimage* 35, 1459–1472. doi: 10.1016/j.neuroimage.2007.02.016.

Tournier, J.-D., Calamante, F., and Connelly, A. (2013). Determination of the appropriate b value and number of gradient directions for high-angular-resolution diffusion-weighted imaging. *NMR Biomed* 26, 1775–1786. doi: 10.1002/nbm.3017.

Tournier, J.-D., Smith, R., Raffelt, D., Tabbara, R., Dhollander, T., Pietsch, M., et al. (2019). MRtrix3: A fast, flexible and open software framework for medical image processing and visualisation. *NeuroImage* 202, 116137. doi: 10.1016/j.neuroimage.2019.116137.

Veraart, J., Fieremans, E., and Novikov, D. S. (2016). Diffusion MRI noise mapping using random matrix theory. *Magnetic Resonance in Medicine* 76, 1582–1593. doi: 10.1002/mrm.26059.

Willats, L., Raffelt, D., Smith, R. E., Tournier, J.-D., Connelly, A., and Calamante, F. (2014). Quantification of track-weighted imaging (TWI): characterisation of within-subject reproducibility and between-subject variability. *Neuroimage* 87, 18–31. doi: 10.1016/j.neuroimage.2013.11.016.

Zigmond, A. S., and Snaith, R. P. (1983). The hospital anxiety and depression scale. *Acta Psychiatr Scand* 67, 361–370.
